# Experiences of midwives on Vscan limited obstetric ultrasound use: a qualitative exploratory study

**DOI:** 10.1186/s12884-022-04523-3

**Published:** 2022-03-10

**Authors:** Mesele Damte Argaw, Hailemariam Segni Abawollo, Zergu Taffesse Tsegaye, Ismael Ali Beshir, Heran Demissie Damte, Birhan Tenaw Mengesha, Zenawork Kassa Gebremedhin, Atrie Fekadu Heyi, Asfaw Adugna Guteta, Tsega Teferi Mamo, Amare Assefa Anara, Zelalem Yilma Emiru, Feyisa Serbessa Yadeta, Almaz Bekele Wami, Mengistu Asnake Kibret, Binyam Fekadu Desta

**Affiliations:** 1 USAID Transform: Primary Health Care Activity, JSI Research & Training Institute, Inc. in Ethiopia, P.O. Box 1392 code 1110, Addis Ababa, Ethiopia; 2 USAID Transform: Primary Health Care Activity, Pathfinder International, Addis Ababa, Ethiopia

**Keywords:** Experiences, Opinions, Midwives, Obstetric ultrasound, Ethiopia

## Abstract

**Background:**

Ethiopia is a low-income country located in the horn of Africa’s sub-Saharan region, with very high incidences of maternal and neonatal mortality. Quality antenatal care improves perinatal health outcomes. The USAID funded Transform: Primary Health Care Activity in collaboration with the Ministry of Health and GE Healthcare introduced Vscan limited obstetric ultrasound services in 120 health centers in Ethiopia. So far, the experiences and opinions of midwives on their use have not been explored and described within the local context. This study therefore aims to explore and describe the experiences and opinions of midwives on Vscan limited obstetric ultrasound services at health centers within Ethiopia.

**Methods:**

An exploratory and descriptive qualitative study was conducted in Amhara, Oromia, and Southern Nations, Nationalities and Peoples’ (SNNP) regions of Ethiopia. Twenty-four participants were selected through a purposeful sampling technique. In-depth individual interviews with trained midwives with practical hands-on limited obstetric ultrasound service provision experience were conducted. The thematic analysis was conducted manually.

**Results:**

The qualitative data analysis on the experiences and opinions of midwives revealed three themes, namely: individual perception of self-efficacy, facilitators, and barriers of limited obstetric ultrasound services. The basic ultrasound training, which was unique in its organization and arrangement, prepared and built the self-efficacy of trainees in executing their expected competencies. Support of health systems and health managers in dedicating space, availing essential supplies, and assigning human resources emerged as facilitators of the initiated limited obstetric ultrasound services, whereas high workload on one or two ultrasound trained midwives, interruption of essential supplies like paper towels, gel, and alternative power sources were identified as barriers for limited ultrasound services.

**Conclusion:**

This study explored the experiences and opinions of midwives who were trained on the provision of limited obstetric ultrasound services and served the community in health centers in rural parts of Ethiopia. The results of this study revealed the positive impacts of the intervention on the perceived self-efficacy, facilitation, and breaking-down of barriers to obstetric ultrasound services. Before scaling-up limited obstetric ultrasound interventions, health managers should ensure and commit to availing essential supplies (e.g., paper towels, ultrasound gel, and large memory hard discs), arranging private rooms, and training other mid-level health professionals. In addition, improving pregnant women’s literacy on the national schedule for ultrasound scanning services is recommended.

**Supplementary Information:**

The online version contains supplementary material available at 10.1186/s12884-022-04523-3.

## Background

Globally, ultrasound examination services for pregnant women has a history that spans about seven decades [1]. In low- income countries where there were limited state-of-the-art machines and qualified sonographers, high-risk conditions of pregnancy were undetected until the time of delivery [2]. Information generated on maternal, fetal, and placental conditions using ultrasound imaging enhances the diagnosis and management of life-threatening conditions [3].

The International Federation of Gynecology and Obstetrics (FIGO) recommends two ultrasound examination services for all pregnant women [4]. Similarly, in 2016, the World Health Organization (WHO), recognized the benefits of offering at least one antenatal ultrasound scanning service, before 24 weeks of gestation for all pregnant women [3, 5]. Introducing an ultrasound scanning service during antenatal care (ANC) is believed to improve the quality of screening, diagnosis, and management of pregnant women. These quality improvements are achieved through the utilization of trained healthcare workers that provide ultrasound services for pregnant women [6, 7]. The health systems in low-income countries are not equipped with the necessary technology and capable human resources to increase access to ultrasound services, [8]. In many countries, including Ethiopia, ultrasound services are mainly available in large urban towns [9]. In addition, the shortage of competent sonographers necessitates the referrals of ultrasound examinations. Therefore, the guidance of offering universal ultrasound scanning services was not feasible for most pregnant women living in rural areas [10].

The USAID Transform: Primary Health Care Activity is a six-year (2017–2022) USAID funded bilateral development cooperative agreement and is currently being implemented in 435 districts in Ethiopia [11]. During its co-creation workshop, the Activity in collaboration with stakeholders identified an innovative technology – Vscan limited obstetric ultrasound – to improve perinatal outcomes [12]. A full set of 100 ultrasound machines were handed over to health centers. In addition, the Activity facilitated 10-day long didactic and practical ultrasound training for 206 mid-level providers who had never been trained on ultrasound usage [11, 13]. Three monthly coaching sessions lasting two days were conducted before certifying the trained providers to run limited obstetric ultrasound scanning services independently in their respective health centers. Furthermore, the health centers were coached to arrange rooms, create demand, and refill essential supplies like ultrasound gel [11].

Several studies confirmed that in low-income countries, the innovation of portable and limited obstetric ultrasound scanning services at the point of care improved maternal and neonatal perinatal outcomes [14–27]. Evidence from studies conducted on Sub-Saharan Africa (SSA) show that antenatal visits and delivery services in healthcare clinics significantly increased after the introduction of low-cost ultrasound programs [15, 23, 25, 27]. Similarly, in Guatemala, a non-randomized interventional observational study of a control group documented evidence of improved perinatal health outcomes [17]. In addition, there is ample evidence on the effectiveness of ultrasound training for the effectiveness of performances of midwives [13, 24, 26]. However, to ensure sustainability of the initiative during task sifting, the commitment and willingness of health workers is one of the critical components of system building. Currently, there is a shortage of evidence on the experiences and opinions of midwives on integration of antenatal ultrasound examinations with routine services at the health center level within a rural setup. To the knowledge of the investigators, there is no published study available on the topic in Ethiopia. It is important to generate context specific evidence which will help policy makers, managers, and development partners shape future large-scale interventions. Therefore, this study aimed to explore and describe the experience and opinions of midwives working at the health center level on innovative Vscan limited obstetric ultrasound integration into routine ANC in Ethiopia.

## Methods and materials

### Study design, setting, and period

An exploratory and descriptive qualitative study design was employed to elicit the experiences and opinions of trained midwives who are providing limited obstetric ultrasound services. This study was conducted in Amhara, Oromia, and SNNP regions of Ethiopia. Delivery case teams offer basic maternal and neonatal healthcare services in health centers and contain mid-level health professionals which include public health officers, nurses, and midwives. Based on the Ethiopian Demographic and Health Survey (EDHS) report of 2019, the proportion of pregnant women who received health services from skilled professionals was 74%, 43%, 48%, and 43% for single ANC, ANC 4 + , skilled birth attendance, and postnatal care services, respectively in the preceding five years [28]. In 2018, the USAID Transform: Primary Health Care Activity, as part of its innovative maternal and neonatal health service improvement interventions, introduced Vscan limited obstetric ultrasound services to 100 health centers in four regional states of Ethiopia [29]. A health center is a health facility at the primary level of the healthcare system which provides promotive, preventive, curative, and rehabilitative outpatient care including basic laboratory and pharmacy services with a capacity for 10 beds for emergency and delivery services. Health centers refer mothers and neonates for cesarean section procedures and for newborn intensive care unit services to nearby primary hospitals [30]. The data for this study were collected from August to December 2020.

### Intervention

The Ministry of Health recruited midwives with no previous experience of scanning, reading, and interpreting ultrasound images and enrolled them into a basic limited obstetric ultrasound training course. The training was facilitated by a group of experts including obstetricians and gynecologists, radiologists, and GE Healthcare technical experts [29]. A ten-day classroom didactic course with orientation on common ultrasound application and practical hands-on Vscan limited ultrasound machines were then arranged. As a part of the syllabus, trainees were expected to continue recording health information, saving images, and providing recommended actions for a period of three months. Trainees received three sessions of monthly onsite and off-site coaching and mentoring. In addition, each trainee was given real-time feedback during the hands-on sessions, and after reviewing the ultrasound images and filled out biodata. At the end of the third month, the trainees’ knowledge was assessed using written exams that required an achievement of a score of greater or equal to 70.0% in order to pass. In addition, each trainee was required to undergo an objective structured clinical examination (OSCE) based skill test, for which a score of 70.0% was needed for certification (additional file 1). Trainees who did not pass both evaluations were provided with a series of one-on-one mentoring sessions and were encouraged to continue self-preparation for re-examination. The evaluated competencies include machine operation, pregnant women preparation, ultrasound imaging, reading, and interpretation. In addition, the trainers assessed and rated each trainee on their ability to identify gestational sac, fetal heart rate, presentation, placenta location and biometry measurement including amniotic fluid, fetal dating, and counseling or communication of trainees with patients. Upon successful completion of the training, the USAID Transform: Primary Health Care Activity donated the Vscan ultrasound machines with seed ultrasound gel, logbooks, and reporting forms [29].

### Sample size and sampling

Twenty-four participants were selected through a purposeful sampling technique. In-depth individual interviews of volunteer trained midwives and nurses with practical hands-on experience on providing limited obstetric ultrasound services were conducted. The final sample size was determined based on data saturation [31]. Data collection ceased when data saturation was achieved, i.e., at the point when ‘no new information or themes were observed in the data’.

### Data collection

The data were collected using pre-tested semi structured in-depth interview guides. The recruited data collectors owned a master’s degree in health sciences or in their professional career and had practical experiences in the whole process of a qualitative inquiry. They were trained for two days on the objectives of the study, data collection techniques, and local and international ethical principles. The experiences and opinions of trained midwives on Vscan limited obstetric ultrasound services at primary healthcare units were explored and described. The two main questions forwarded to midwives were: (1) ‘Would you tell me your views on the role of Vscan limited obstetric ultrasound services on management of maternal and neonatal healthcare services?’ and (2), ‘How do you assess your skill and experience on the provision of Vscan limited obstetric ultrasound scanning services?’. The in-depth interviews were conducted using the local official languages in the study areas, that is, Amharic in Amhara and SNNP regions, and Afan Oromo in the Oromia region. Digital Audio recorders (SONY ICD -PX333) were used to capture all data and audio files were transcribed and translated by the investigators, and were analyzed manually.

### Data analysis

The qualitative data were transcribed verbatim and translated into the English language. The data analysis process followed six phases of thematic analysis. The first phase was reading and re-reading all the transcripts multiple times for the investigators to become immersed and familiarized with the contents. The second phase was labeling the identified seventy-five codes in the whole document and collating extracts associated with each code. The third phase was generating categories based on broader patterns of the collated data. The fourth phase was reviewing, merging, and deleting categories. The fifth phase was a detailed analysis of themes, where the investigators compared and agreed on the interpretations. The sixth phase was writing up the results within the context of available literature.

### Measures to ensure trustworthiness

The qualitative study was conducted by a team of researchers including gynecologists/obstetricians, public health experts, and health systems strengthening specialists. The in-depth interviews were conducted using a pretested semi-structured guide. The experiences and opinions of the research participants who successfully completed the limited obstetric ultrasound training and had served the community for more than two years at the health centers level were described using their own words. To ensure trustworthiness, two investigators separately coded and categorized the data. Consensus meetings were organized, and themes were identified together. Participants were offered the opportunity to review the themes, categories, and quoted verbatims as a member check. Furthermore, Lincoln and Guba’s five criteria of trustworthiness (credibility, transferability, dependability, confirmability, and reflexivity) were maintained [32, 33]. The credibility of this study was maintained through utilization of direct quotes of participants and their participation in the validation of the interpretations as a member check. The transferability of this study was ensured using a thick description of the methods employed in this study. The dependability of this study was maintained through engagement of participants in the analysis processes. The confirmability of this study was ensured through a step-by-step data collection, analysis, and interpretation process as clearly stated in the ‘methods’ section. The reflexivity of this study was maintained through assigning experienced and trained qualitative data collectors. In addition, the data analysis was independently conducted by two investigators.

### Ethical considerations

This study was carried out in accordance with the Declaration of Helsinki and ethical clearance was granted by the JSI Research & Training Institute, Inc. institutional review board (IRB #20-26E). A brief orientation on the purpose and objectives of the study was given to all participants of this study. Participants also signed a written informed consent to be interviewed and consented to being audio recorded. Participants were informed that they have the right to discontinue or refuse to participate in the study at any time. The investigators maintained the confidentiality and anonymity of participants and employed ethical practices throughout the whole process of the study.

## Results

### Participant characteristics

This exploratory qualitative study was conducted through 24 in-depth interviews of midwives, who were trained to provide antenatal ultrasound services at the health center level in rural setups in Ethiopia. The majority (18/24) were female, and all (24/24) were midwives by profession. The age of the participants ranged from 24 to 38 years. In addition, the tenure of each interviewee averaged 6.6 and 2.5 years for health system and ultrasound scanning services, respectively.

The data analysis identified three themes, which include individual perception on self-efficacy, facilitators, and barriers of limited obstetric ultrasound services.

### Theme 1: individual perception on self-efficacy

The participants of this study asserted that the nature and mixed method organization, including didactic, practical hands-on limited obstetric ultrasound training, review meetings, experience sharing events, and feedback through phone applications enabled them to believe in their scanning, diagnosing, and confirming capacities and capabilities. This theme emerged from four categories: ultrasound training, the work of midwives, pregnant women’s expectation, and health service quality.

### Category 1.1. ultrasound training

The limited obstetric ultrasound training was conducted by experienced sonographers, who are radiologists and gynecologists/obstetricians. The trainees were mid-level health professionals, (mainly midwives), recruited from targeted health centers. The facilitation was carried out by the Ministry of Health and its developmental partners. A ten-day didactic course and practical hands-on sessions of limited ultrasound machines were arranged in health centers where adequate cases were found. In addition, three subsequent offsite coaching and mentoring sessions were organized for consecutive months which were supplemented with continuous virtual feedback provision through phone application groups which made the training unique. Trainees were certified to continue independently upon successful completion of the training.

The limited ultrasound training mainly focused on normal anatomy, measuring fetal biometry including mean sac diameter (MSD), crown-rump length (CRL), biparietal diameter (BPD), head circumference (HC), abdominal circumference (AC) and femur diaphysis length (FL), and estimating amniotic fluid level. In addition, it helped the mid level professionals to identify fetal cardiac activity, fetal position (cephalic, breech, transverse), fetal numbers, placenta location (previa, low lying, fundal), and diagnose fetal growth disorder (SGA or LGA). Furthermore, using the measurement reports, trainees were expected to generate tailored information for pregnant women, mainly on their Estimated Data of Delivery (EDD), abnormalities (if any were found), and assist mothers in the development of a management plan.

Most participants of this study reported the ultrasound training as useful and said it built on their knowledge, skills, and confidence in providing standard prenatal services. In addition, almost all participants stated that the training was unique and motivated them to provide ultrasound scanning services on weekdays, during night shift, and on weekends. A midwife from a rural health center had this to say:

*“The ultrasound training improved my knowledge and competencies which were necessary in identifying high-risk health conditions for both the women and their fetuses… Before this, I worried that I was not competent and might miss critical conditions in patients. The training built up my confidence in screening, diagnosing, and holding discussions with pregnant women.”*

Another in-depth interview participant described her improved confidence on ultrasound application as follows:

*“I am confident in my surveying of the uterus and ovaries of patients. I can also generate a clear image of intrauterine pregnancy, cysts, suspected fluids, and retained products of conceptions. In addition, I have perfected measuring the fetal biometry parameters including crown-rump length, amniotic sac diameter, and yolk sac size, identifying fetal position, multiple fetuses, placental locations, fetal cardiac activity, and gross anomalies. The knowledge and skills I gained motivates me to provide the ultrasound services during weekdays, weekends, holidays, and during night shift hours .”*

A third participant explained the uniqueness of the basic ultrasound training by saying:

*“The ultrasound training, which I took part in, was unique in its organization and delivery modality. The trainers were skilled gynecologists or obstetricians, and after basic training and demonstration in a classroom setting, we treated pregnant women under their supervision. In addition, we continued our hands-on practice for three months. We shared our saved ultrasound images and reports with our trainers using a Telegram phone application group and feedback was given in real time. In addition, we underwent three off-site mentoring and coaching sessions before certification. The training manual I was given was colorful and a useful reference material for my day-to-day ultrasound service tasks.”*

### Category 1.2. work of midwives

According to the participants of this study, midwives are expected to provide routine maternal, neonatal and child health services in health centers. In many health centers, there are two or three midwives who are responsible for providing complete and comprehensive health services. Some of the standardized services expected from midwives are ANC, family planning, skilled birth attendances, newborn care, postpartum care, and integrated management of childhood illness services. In addition, they work on weekdays, during night shift hours, weekends, and on emergency services. Furthermore, the newly introduced limited obstetric ultrasound services demand much more time with each pregnant woman, to screen and diagnose maternal or fetal health conditions. The ultrasound scanning report is used to inform pregnant women and arrange referral services for high-risk conditions. Furthermore, the ultrasound machine/probe, examination couches, vital sign equipment, and the treatment rooms need disinfection and cleaning before examinations. A participant explained additional responsibilities she acquired on top of to her regular duties after the introduction of ultrasound, saying:

*“My colleague and I are responsible for providing all maternal and child health services, 24 hours a day and seven days a week in our health center. After I participated in the ultrasound training, I had additional tasks added on. Each pregnant woman now needs about 20 minutes for a single scanning service, and I register and record the findings on the ultrasound logbook. I also facilitate counseling and planning sessions based on the created ultrasound report. In addition, it is my responsibility to clean the examination couch, vital sign measuring equipment, examination room, and the ultrasound probe/transducer.”*

Another midwife had this to say about her follow-up tasks for some pregnant women who had been identified as potentially having a high-risk health condition:

*“During the second trimester if I diagnose a fetal transverse position, I reassure the pregnant woman that it’s not a reason for worry and continue her follow-up care. It is only if the condition continues into the late stages of the third trimester that I will refer pregnant women to the nearest primary hospital.”*

### Category 1.3. pregnant women’s expectations

The antenatal ultrasound training is designed to capacitate mid-level health workers to provide limited obstetric screening, confirming, and diagnostic services at least once—preferably during the second trimester of pregnancy. Nevertheless, pregnant women can demand ultrasound scanning services to confirm pregnancy at as early as four weeks. In addition, pregnant women’s desire to know the sex of the fetus, presentation, and any abnormalities is high. A participant stated the expectation of pregnant women which is beyond her competencies by saying:

*“Pregnant women visit our health center to confirm their pregnancy and expected date of delivery. They want to know the sex of their fetus, but we were not taught about how we can find this out. In addition, if we find any high-risk conditions for both pregnant women and their fetus, we are expected to report and arrange referral services for them. Some women also want to have a written ultrasound report, but we do not have the capacity to print it and give it to them.”*

### Category 1.4. quality prenatal health services

Almost all participants stated that the introduction of the ultrasound technology at the health center level improved perinatal health outcomes. In addition, the fee exempted limited obstetric ultrasound services incurred no additional costs for pregnant women and increased eligible women’s referral and utilization of life-saving services. Furthermore, pregnant women were grateful for the ultrasound services and the detailed information given to them by the midwives. An in-depth interview participant expresses the effects of ultrasound machines on the quality of perinatal services stating:

*“The [ultrasound] we provided creates a nice image and report which facilitated my communication with pregnant women. The service is safe for pregnant women and is offered free of charge. I identified many pregnant women with multiple fetuses, breech or transfer presentations, and low-lying placenta which I referred to primary hospitals. I am very happy that through this I was able to contribute to reducing maternal and neonatal deaths among my catchment community. In addition, pregnant women are satisfied with these services.”*

A midwife recalled a high-risk health condition she identified with the help of the ultrasound machine for which she was able to arrange a referral service, saying:

*“The ultrasound scanning helped me to identify pregnant women with confirmed diagnoses of blighted ovums, gestational trophoblastic disease, metastasis, missed abortions, and ectopic pregnancies. These diagnoses at our rural health center were verified by the referral receiving facilities.”*

Another midwife explained that the ultrasound is regarded highly by health professionals and pregnant women alike as she described just how critical it is for the management of pregnant women:

*“We call our ultrasound the ‘mother of mothers.’ Without it we would have difficulty with correct diagnosis and would struggle to recommend the right management at the right time. It helps us to diagnose pregnancy, its location and estimate date of delivery during the first trimester, fetus position, health status, placenta location, amniotic fluid level, and gross abnormalities in the second trimester. Therefore, the service is key to achieving reductions in maternal and neonatal deaths.”*

A respondent also explained that the presence of ultrasounds alleviates the stress in midwives, saying:

“The availability of the limited obstetric ultrasound services at our rural health center alleviates stress in midwives and improves perinatal health outcomes.”

### Theme 2: facilitators of the limited obstetric ultrasound services

According to the participants, there were facilitators of the limited obstetric ultrasound service provision at the health center level. This theme emerged from categories which described community acceptance, health system support, and development partner support.

### Category 2.1. acceptance of communities

Communities were informed of the availability of ultrasound services through pregnant women’s conferences, health extension workers, and voluntary village health leaders. Almost all participants stated that the acceptance and demand for the ultrasound services by the community were very high. An in-depth interview participant explained that ANC service beneficiaries increased after introducing ultrasound services claiming:

*“The introduction of ultrasound services in [name] health center was included in the discussion agenda of pregnant women conferences which were facilitated at the village level. Though I am expected to provide ultrasound scanning service for women during 18 to 22 weeks of gestation, many pregnant women requested their ANC checks before 12 weeks. Therefore, after the introduction of the ultrasound machine, maternal and child health service utilization increased.”*

### Category 2.2. health system support

Most of the participants described their clear understanding of the national health strategic directions and testified on the commitment of the Ministry, through offering service fee exempted obstetric ultrasound scanning service for each pregnant woman. In addition, they asserted that the introduction of ultrasound technology helped the midwives provide good quality services to pregnant women. A midwife described the support she received from the health center as:

*“The health center trained and assigned me to provide ultrasound services and development partners donated the Vscan ultrasound machine with seed supplies. The health center dedicated a space in the maternal and child health room where the service is offered for free. Our health center bought essential supplies to ensure the continuity of the ultrasound services.”*

### Category 2.3. development partners’ support

The research participants stated their opinions on the role of development partners facilitators for the innovative limited obstetric ultrasound services in Ethiopia. Most of the participants explained that the ultrasound machine, training, coaching, and mentoring sessions held by senior experts, collecting and analyzing of data, and organization experience sharing events were implemented with the support of development partners. A midwife expresses her opinion on the role of development partners as:

*“[Name] – a development partner donated the Vscan ultrasound machine, and I attended the training and coaching sessions. I was also sponsored by [name] to participate in knowledge sharing events, where trainees presented their stored images and reports. Partners recognize and motivate midwives which ensures continued ultrasound services.”*

Despite this, one of the in-depth interview participants claimed that the limited ultrasound training and the skills he acquired did not help to identify some fetal anomalies. He stressed the importance long-term training by development partners, saying:

*“After informing a pregnant woman that her fetus is normal, the baby was born with clubfoot. I was ashamed of my diagnosis. I would be happy to attend a subspecialty training in sonography. If the development partners organize a long-term ultrasound training, it will immensely help my career development in the area.”*

### Theme 3: barriers of the limited obstetric ultrasound services

According to the reported experiences and opinions of research participants, there were several barriers to optimally implementing the initiated limited obstetric ultrasound services at the health center level. The third theme identified was related to barriers of ultrasound services which emerged from three categories, namely, infrastructure, essential supplies, and workload.

### Category 3.1. lack of infrastructure

Lack of infrastructure including unavailability of private rooms and alternative electric power sources were repeatedly mentioned by more than half of the participants as major reasons for the interruption of the limited ultrasound services in health centers. A participant explained the difficulties she faced in maintaining the privacy of pregnant women, saying:

*“Only three rooms are allocated for all maternal and child health services in our health center. It is difficult for me to provide ultrasound scanning services while taking the necessary precautions for maintaining privacy. The initiated antenatal ultrasound service for pregnant women requires the assigning of a separate room.”*

Another participant described the interruption of electric power as a main barrier for obstetric ultrasound services, saying:

“The [name] health center does not have an alternative electric power source; the ultrasound service relies on the availability of a high-grade electric power supply.”

### Category 3.2. lack of essential supplies

The limited obstetric ultrasound service needs supplies like tissue paper, ultrasound gel, memory sticks, referral forms, and uninterrupted power source or stabilizers. Almost all research participants asserted that the interruption of essential supplies demotivated midwives to continue with the provision of the ultrasound services. A midwife described the interruption of ultrasound services as a result of lack of essential supplies, saying:

*“I remind the health center management several times to purchase ultrasound gel and tissue papers for me. When this doesn’t happen, the ultrasound scanning service can be interrupted for up to two to three months.”*

Another midwife explained the similar challenges she faced as follows:

*“When our health center does not refill tissue paper stock, I use gauzes in order not to interrupt the ultrasound services. But there were times when pregnant women were forced to return to their home without receiving [ultrasound] services due to shortage of supplies. This frustrates me and puts me off from resuming the provision of ultrasound services.”*

A midwife described how a low memory storage stick he was provided with failed to save important information and bio-data as follows:

*“The Vscan ultrasound machine only has 4 GB of internal memory. It cannot save large numbers of images and pregnant women’s health information. Although I have tried to export the data to my computer, it wasn’t readable. I requested the purchase of a larger size external memory stick by the health center but have not been provided with one.”*

### Category 3.3. workload

The participants of this study frequently pointed out that the number of antenatal ultrasound service beneficiaries would increase from time to time. They said that the additional tasks they were assigned after the introduction of the ultrasound machines in their health centers obliged them to provide services 24 h a day, 7 days a week. A midwife explained the added workload after the introduction of an ultrasound machine in her health center by saying:

*“As the limited obstetric ultrasound service was initiated in a few health centers, I was not expecting the demand for the provision of services to be so high among our catchment population. But beneficiaries continually increased. Due to the workload, it is becoming difficult for a trained midwife to provide ultrasound services as part of routine services.”*

Another midwife talked about the interruption of services she experienced while on duty, saying:

“If I am on duty during the night, I will not be available to provide services in the daytime. Therefore, with the exception of emergency cases, ultrasound services will not be given.”

A midwife stated that after her colleague was transferred to the district’s health office, she was left to provide the services alone, saying:

*“In [name] health center, we were working as two ultrasound trained midwives. My colleague was then transferred to the district’s health office as a maternal and child health core process owner. Now, it is only me that can provide ultrasound scanning services in the health center.”*

## Discussion

The introduction of antenatal ultrasound services at the rural health center level in Ethiopia has improved the adherence of pregnant women to ANC visits and has also improved the perinatal health outcomes through early diagnosis and management of high-risk health conditions. Based on global recommendations, the Ethiopian Ministry of Health, through its strategic plan (2020/21–2024/25) is working to offer at least one ultrasound service for all pregnant women before 24 weeks of gestation [34]. However, during the last five years, the public health sector led antenatal ultrasound services were only available in referral hospitals and a few health centers. This exploratory and descriptive qualitative study aimed to capture the experiences and opinions of 24 obstetric ultrasound operation trained mid-level providers, in three regional states of Ethiopia. The results of the study identified three main themes including individual perception on self-efficacy, facilitators, and barriers of the initiated limited obstetric ultrasound services. These findings will help stakeholders to take advantage of the perceived qualities of the ultrasound training and strategize to solve infrastructure and essential supply related challenges during future similar interventions.

It was the perception of most of the study participants that they could operate the limited obstetric ultrasound and generate useful information for evidence-based decision-making, referral service arrangement, and continuation of follow-up services within the health center. The participants also stated that their experiences of the ultrasound training was innovative and met the expected quality. This could be explained by the mixed nature of the training which includes a didactic course and practical hands-on sessions, as well as a series of coaching sessions by experts. In addition, the provision of real-time feedback during hands-on practical coaching sessions and established virtual platforms might improve the competencies, motivation, and commitment of trainees in providing the limited obstetric ultrasound services to pregnant women. This finding was consistent with Westerway (2019) and Hall et al., (2021) who relate the effectiveness of ultrasound training programs with the experiences of trainers and high level of commitment of trainees [19, 35]. In addition, Swanson et al., (2016) documented that a web-based ultrasound image revision and feedback mechanism were employed to build the capacity of trainees [36].

The introduction of the antenatal ultrasound services has added recording, counseling, and arranging of referral services on the work of midwives. This is the result of the health system’ efforts to mobilize large numbers of pregnant women who reside within the catchment area of health centers. However, this mass mobilization is not in accordance with the Ministry of Health’s recommended approach of providing at least one ultrasound service for a pregnant woman before 24 weeks of gestation. In addition, pregnant women may demand ultrasound scanning services beyond the frequency stated in the minimum standards [27]. This finding was consistent with the course syllabus and expected competencies from certified trainees. Mbuyita et al., (2015) revealed that in Tanzania, after the introduction of ultrasound services, the number of fourth ANC service beneficiaries doubled from the baseline figure [16]. Similarly, Ross et al., (2018) attest that the innovation was linked to increased ANC clinic visits and attendance of skilled births in Uganda [15].

Participants of this study agreed on the expectations of pregnant women from the ultrasound services, which include confirming the pregnancy, their health condition, fetal growth, and development status. In addition, pregnant women want to avoid fetal abnormalities. This finding is consistent with Alyahya et al., (2019) on the reason for seeking ANC which includes avoiding fetal abnormalities, pregnancy related complications, and receiving multivitamins [37]. However, some midwives reported being frustrated with the demand of pregnant women to find out the sex of their fetus. This could be explained with the limited nature of obstetric ultrasound training which does not include fetal sex determination. Kim et al., (2018) through a narrative review confer that women demand to know sex of their fetus despite it being illegal to determine the sex of the fetus in many counties [8].

Antenatal, intrapartum, and postpartum complications are among the most common causes of maternal and neonatal deaths [37]. This study revealed that the integration of antenatal ultrasound services had improved the quality of prenatal health services. This could be achieved due to the created capacity and capability of midwives to detect and forecast serious health conditions with the help of ultrasound. In addition, the introduction of this technology may enhance standardized and step by step care. Furthermore, this effective and efficient use of technology can enhance client safety, equity, and timely provision of client centered services. This finding is in line with the reviewed chapter of Wiafe et al., (2011) on the role of ultrasound in reducing maternal and neonatal mortality [38].

The opinions of participants showed that there were high levels of acceptance of the initiated ultrasound service by community members and it had encouraged them to provide services. This finding was in line with the pilot study of Amoah et al., (2016) who aligned boosting of ANC attendance with implementing mobile phone applications and portable ultrasound scanning in Ghana [39]. In addition, the presence of health system support including assigning personnel, dedicating ultrasound service space, and refilling of essential supplies were the facilitators of the ultrasound service at health center level. Furthermore, the existence of development partners was a facilitator for the limited ultrasound innovation through the donation of the ultrasound machines with seed supplies, and preparation and motivation of service providers at health center level [40]. The findings were consistent with Swanson et al., (2017) who confer the importance of willingness and commitment of health system management to facilitate ultrasound services in a pilot district in the Democratic Republic of the Congo and Rwanda [7].

The participants of this study identified barriers of the limited ultrasound service implementation which include lack of ultrasound private rooms and interruption of electric power sources. Likewise, almost all participants frequently mentioned that shortage of ultrasound gel, paper towels, and large sized memory sticks were barriers to the initiated ultrasound service. This finding was in line with the field report of Swanson et al., (2017) where they clearly describe inaccessibility to continuous electric supply and incomplete set of alternative power sources as a challenge for ultrasound services in low-income countries [7].

With the current high workload experienced by midwives, the ultrasound service could not be offered to all pregnant women who visited the health centers during weekdays. This could be explained by the routine maternal, neonatal and child health services in addition to the integrated ultrasound examination services being provided by a single or two trained midwives. Campbell (2013) confer that the implementation of ultrasound for ANC in low-resource settings has been shown to increase pregnant women’s compliance with ANC visits and facilitate the detection of high-risk pregnancies [1].

### Strength and limitations

This qualitative study explored the experiences and opinions of midwives who provide limited obstetric ultrasound services in health centers in Ethiopia. Since, the use of portable ultrasound service at point of care is expected to grow exponentially in the coming decades, learning from early adopters helps stakeholders to maintain, design, and redesign interventions. This study enrolled participants from three regional states of Ethiopia. This enabled the investigator to explore and document the experiences of midwives working in a wider context, within a decentralized health system in Ethiopia. This study has some limitations including that it does not incorporate the experiences and opinions of direct service beneficiaries and supervisors of the study participants. In addition, the effects and contributions of introducing limited obstetric ultrasound on service utilization and health outcome were not documented in this article.

## Conclusions

This study documented the experiences and opinions of midwives who are competent and provide limited obstetric ultrasound scanning services in health centers within Ethiopia. The study uncovered midwives’ positive outlook about their ability of ultrasound operating, scanning, imaging, reading, interpreting, and documenting of maternal and fetal health conditions. The unique nature of using a didactic course supplemented with practical hands-on coaching sessions with real time feedback provision enhanced the motivation and commitment of midwives to serve their rural communities. The intervention also improved the screening, diagnosing, and counseling skills of midwives so as it improved perinatal health outcomes. Support from the health system and health managers in availing essential supplies was identified as a facilitator for the limited obstetric ultrasound services. However, several barriers to the service emerged in this study which included expectations of mothers beyond the capacity of midwives, shortage of supplies like gel, report forms, and external hard discs, as well as interruption of electric power sources. Therefore, before scaling-up the limited obstetric ultrasound intervention, health managers should ensure and commit to availing essential supplies (paper towels, ultrasound gel, and large memory hard discs), arrange private rooms, and train other mid-level health professionals. In addition, improving pregnant mothers’ literacy on the national schedule for ultrasound scanning services is recommended.

## Supplementary Information

**Additional file 1.** Observed StructuredClinical Exam Assessment Form.

## Data Availability

All relevant data are within the paper.

## Abbreviations

AC: Abdominal Circumference
ANC: Antenatal Care
BPD: Biparietal Diameter
CRL: Crown-rump Length
EDD: Expected Date of Delivery
EDHS: Ethiopian Demographic and Health Survey
FL: Femur Diaphysis Length
FP: Family Planning
HC: Head Circumference
LGA: Large for Gestational Age
MSD: Mean Sac Diameter
PNC: Postnatal Care
FIGO: International Federation of Gynecology and Obstetrics
SGA: Small for Gestational Age
SNNP: Southern Nations, Nationalities, and Peoples’
USAID: United States Agency for International Development
WHO: World Health Organization
