# Supplementary material for: Experiences of midwives on Vscan limited obstetric ultrasound use: a qualitative exploratory study
Source: BMC Pregnancy Childbirth. 2022 Mar 10;22:196. doi: 10.1186/s12884-022-04523-3 (PMC8915526; doi:10.1186/s12884-022-04523-3)
Supplement: Supplementary file 1 — Additional file 1. Observed StructuredClinical Exam Assessment Form. [file 12884_2022_4523_MOESM1_ESM.docx]

**Observed Structured Clinical Exam Assessment Form**

**Trainee Name: ____________________________________________________________**

**Designation: ______________________________________________________________**

**Date: ____________________________________________________________________**

Evaluator Name: ___________________

Student Name: ____________________ Date of Assessment: ____________

| Site Assessment and Workflow | | **Evaluation (Y/N)** |
| --- | --- | --- |
| 1 | Scan room Available |  |
| 2 | Scanning Coach |  |
| 3 | Power available |  |
| 4 | Consumable available |  |
| 5 | Dedicated scan time scheduled |  |
| 6 | Dedicated scan days scheduled |  |
| 7 | ANC Attendance logged |  |
| 8 | Number of Deliveries logged |  |
| 9 | Supporting Nurse available |  |
| 10 | Mentor /Referral facility established |  |

**Comments/Mentoring Needs:**

Evaluator Name: ___________________

Student Name: ____________________ Date of Assessment: ____________

**Scoring:**

0 – Not Acceptable

1 – Needs Improvement

2 – Good/Satisfactory

3 - Excellent

| **Pelvic and 1^st^ Trimester Competency Checklist** | | **Score (0,1,2,3)** |
| --- | --- | --- |
| 1 | ***Patient Introduction / Gender Disclosure Policy** |  |
| 2 | Patient Preparation (clothing, gel, positioning) |  |
| 3 | ***Probe Orientation Notch** |  |
| 4 | Image Optimization (gain, depth, Auto IQ) |  |
| 5 | Survey Uterus / Adnexal region / Cul-de-sac |  |
| 6 | ***Right Ovary (adnexal region if not visible)** |  |
| 7 | ***Left Ovary (adnexal region if not visible)** |  |
| 8 | ***Uterus Length** |  |
| 9 | ***Image Confirming Intrauterine Pregnancy (vs. ectopic)** |  |
| 10 | ***Crown Rump Length – CRL (when embryo visualized)** |  |
| 11 | ***Cardiac Activity (visualized)** |  |
| 12 | Storing / Exporting Images & Report |  |
| 13 | ***Minimum Report Notes:**  **1. Pregnancy Location (*intrauterine pregnancy*)**  **2. Abnormal Findings (*cyst on right ovary, free fluid, suspected ectopic pregnancy, CRL not obtained, etc.*)** |  |
| 14 | End Examination |  |
| 15 | Patient Instructions (EDD, follow-up, referral) |  |
| 16 | Equipment Cleaning, Disinfecting, Hand Washing |  |

*** Items asterisked = critical concepts proficiency must be demonstrated for successful course completion.**

**Comments/Mentoring Needs:**

Evaluator Name: ___________________

Student Name: ____________________ Date of Assessment: ____________

**Scoring:**

0 – Not Acceptable

1 – Needs Improvement

2 – Good/Satisfactory

3 - Excellent

| **2^nd^/3^rd^ Trimester Competency Checklist** | | **Score (0,1,2,3)** |
| --- | --- | --- |
| 1 | ***Patient Introduction / Gender Disclosure Policy** |  |
| 2 | Patient Preparation (clothing, gel, positioning) |  |
| 3 | ***Probe Orientation Notch** |  |
| 4 | Image Optimization (gain, depth, Auto IQ) |  |
| 5 | Survey Uterus / Adnexal region / Cul-de-sacs |  |
| 6 | ***Fetal Number (singleton, multiples)** |  |
| 7 | ***Fetal Position (cephalic, breech, transverse)** |  |
| 8 | ***Amniotic Fluid Level (↑ or ↓ fluid)** |  |
| 9 | ***Cervical Length (competent, funneling)** |  |
| 10 | ***Placental Location (previa, low-lying, fundal)** |  |
| 11 | ***Bi-Parietal Diameter (BPD)** |  |
| 12 | Head Circumference (HC) |  |
| 13 | 4-Chamber Heart/Situs (include T-spine) |  |
| 14 | ***Cardiac Activity (visualized)** |  |
| 15 | ***Abdominal Circumference (AC)** |  |
| 16 | Bilateral Kidneys (visualized) |  |
| 17 | ***Abdominal Umbilical Cord Insertion** |  |
| 18 | Lumbar Spine – Transverse |  |
| 19 | Bladder |  |
| 20 | ***Femur Length (FL)** |  |
| 21 | Storing / Exporting Images & Report |  |
| 22 | ***Minimum Report Notes:**  **1. Placental Location (*anterior, fundal, previa, etc.*)**  **2. Abnormal Findings (*decreased amniotic fluid, BPD/HC not obtained due to patient body habitus/fetal position, etc.)*** |  |
| 23 | End Examination |  |
| 24 | Patient Instructions (EDD, follow-up, referral) |  |
| 25 | Equipment Cleaning, Disinfecting, Hand Washing |  |

*** Items asterisked = critical concepts proficiency must be demonstrated for successful course completion.**

**Comments/Mentoring Needs:**
